# INDEED–Utilization and Cross-Sectoral Patterns of Care for Patients Admitted to Emergency Departments in Germany: Rationale and Study Design

**DOI:** 10.3389/fpubh.2021.616857

**Published:** 2021-04-16

**Authors:** Antje Fischer-Rosinský, Anna Slagman, Ryan King, Thomas Reinhold, Liane Schenk, Felix Greiner, Dominik von Stillfried, Grit Zimmermann, Christian Lüpkes, Christian Günster, Natalie Baier, Cornelia Henschke, Stephanie Roll, Thomas Keil, Martin Möckel

**Affiliations:** ^1^Emergency and Acute Medicine (Charité Virchow Klinikum-CVK, Charite Campus Mitte-CCM), Charité–Universitätsmedizin Berlin, Berlin, Germany; ^2^Institute of Social Medicine, Epidemiology and Health Economics, Charité–Universitätsmedizin Berlin, Berlin, Germany; ^3^Institute of Medical Sociology and Rehabilitation Science, Charité–Universitätsmedizin Berlin, Berlin, Germany; ^4^Department of Trauma Surgery, Otto von Guericke University Magdeburg, Magdeburg, Germany; ^5^Central Research Institute for Ambulatory Health Care in Germany (Zi), Berlin, Germany; ^6^TMF–Technology, Methods, and Infrastructure for Networked Medical Research, Berlin, Germany; ^7^OFFIS–Institute for Information Technology, Oldenburg, Germany; ^8^Allgemeine Ortskrankenkasse (AOK) Research Institute–Wissenschaftliches Institut der AOK (WIdO), Berlin, Germany; ^9^Kiel Institute for World Economy, Kiel, Germany; ^10^Department of Health Care Management, Berlin University of Technology, Berlin, Germany; ^11^Faculty of Health Sciences Brandenburg, Brandenburg University of Technology Cottbus-Senftenberg, Cottbus, Germany; ^12^Institute for Clinical Epidemiology and Biometry, University of Würzburg, Würzburg, Germany; ^13^State Institute of Health, Bavarian Health and Food Safety Authority, Bad Kissingen, Germany

**Keywords:** emergency department, routine health care data, cross-sectoral data analysis, inadequate utilization, ambulatory care sensitive conditions

## Abstract

**Introduction:** The crowding of emergency departments (ED) has been a growing problem for years, putting the care of critically ill patients increasingly at risk. The INDEED project's overall aim is to get a better understanding of ED utilization and to evaluate corresponding primary health care use patterns before and after an ED visit while driving forward processes and methods of cross-sectoral data merging. We aim to identify adequate utilization of EDs and potentially avoidable patient contacts as well as subgroups and clusters of patients with similar care profiles.

**Methods:** INDEED is a joint endeavor bringing together research institutions and hospitals with EDs in Germany. It is headed by the Charité–Universitätsmedizin Berlin, collaborating with Otto von Guericke University Magdeburg, Technische Universität Berlin, the Central Research Institute of Ambulatory/Outpatient Health Care in Germany (Zi), and the AOK Research Institute as part of the Federal Association of AOK, as well as experts in the technological, legal, and regulatory aspects of medical research (TMF). The Institute for Information Technology (OFFIS) was involved as the trusted third party of the project. INDEED is a retrospective study of approximately 400,000 adult patients with statutory health insurance who visited the ED of one of 16 participating hospitals in 2016. The routine hospital data contain information about treatment in the ED and, if applicable, about the subsequent hospital stay. After merging the patients' hospital data from 2016 with their outpatient billing data from 2 years before to 1 year after the ED visit (years 2014–2017), a harmonized dataset will be generated for data analyses. Due to the complex data protection challenges involved, first results will be available in 2021.

**Discussion:** INDEED will provide knowledge on extracting and harmonizing large scale data from varying routine ED and hospital information systems in Germany. Merging these data with the corresponding outpatient care data of patients offers the opportunity to characterize the patient's treatment in outpatient care before and after ED use. With this knowledge, appropriate interventions may be developed to ensure adequate patient care and to avoid adverse events such as ED crowding.

## Introduction

Emergency departments (EDs) in many countries face the challenge of crowding and increasing numbers of ED visits (1). The number of ED visits has increased over the last decades in almost all OECD countries (2). Annually around 21 million patients are treated in German EDs (3). From 2009 to 2015 the number of patients in EDs increased by 42% for outpatient care while inpatient emergency cases grew by 20% (4). One reason for the rising ED patient numbers in outpatient treatment is the utilization of the EDs' 24/7 available medical expertise for minor health problems. This is due to several reasons, with patients often citing health anxiety and lack of alternatives (5–7). More than 50% of ED patients do not require subsequent hospital inpatient treatment, but approximately only 20% could have been treated in the outpatient sector/outpatient care (8, 9). Care provision for patients with low urgency health needs in the emergency setting is currently being heavily debated as hospital-based outpatient care is associated with high costs and does not offer the same continuity as primary care in the outpatient sector (10). Additionally, a large share of acute care patients receiving inpatient treatment after an ED visit are diagnosed with Ambulatory Care Sensitive Conditions (ACSC), i.e., frequent chronic and acute diagnoses for which inpatient care could have been avoided by timely and adequate measures in the outpatient care sector (11–14).

Therefore, the INDEED-project (Utilization and cross-sectoral patterns of care for patients admitted to emergency departments in Germany) will (1) explore the utilization and cross-sectoral patterns of care for patients admitted to EDs in Germany, and (2) provide a framework for future data linkage that takes into account the ethical, legal, and technological aspects for creating a unique data set by merging data from different sectors over a time period of 3 years.

## Methods

### Design

**Figure 1:**
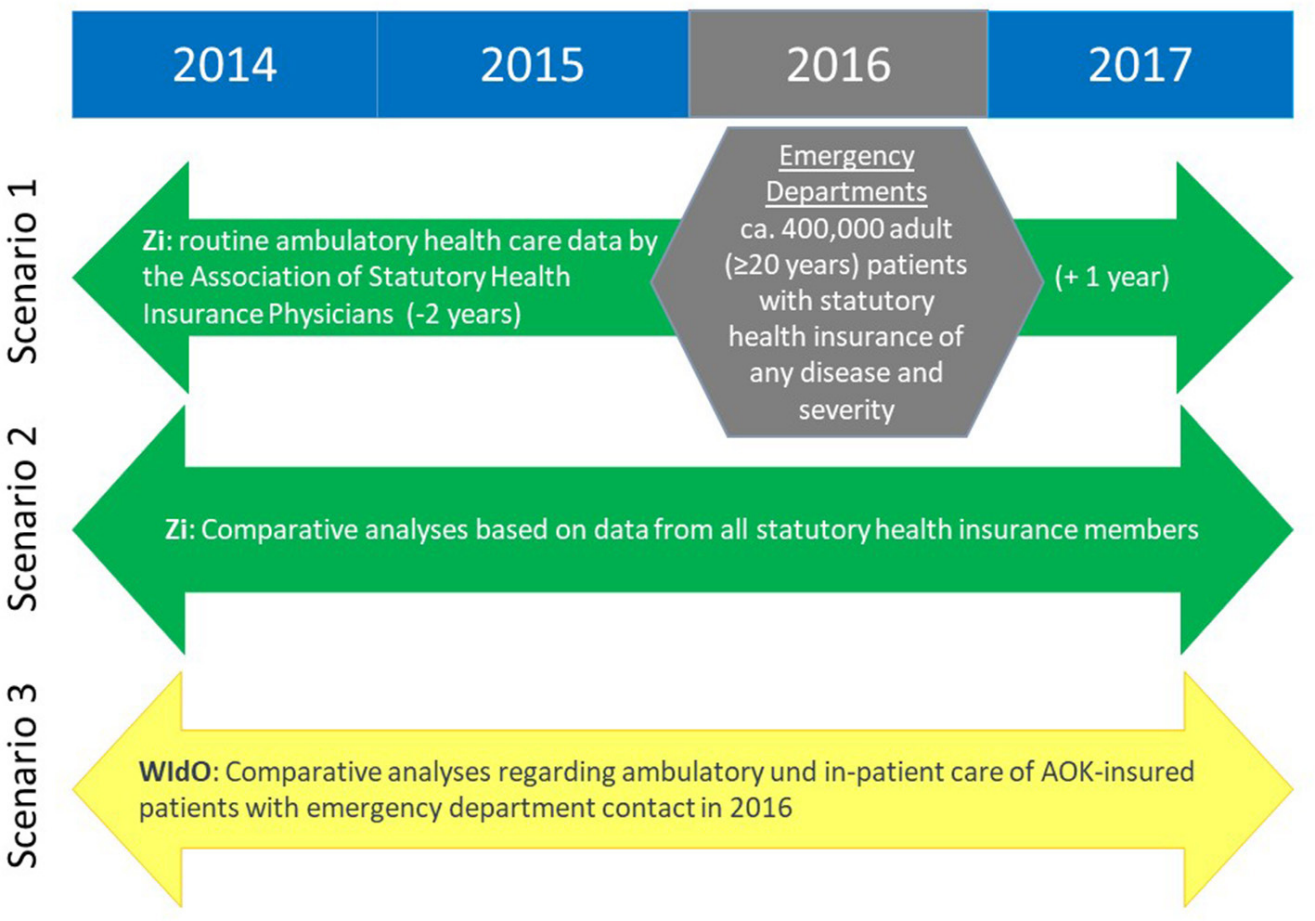
Project scenarios 1–3 using the evaluation of different data sources.

INDEED is designed as a retrospective evaluation of three different scenarios based on different data sources (Figure 1), with the aim of illustrating ED use in 2016. The main research question focuses on scenario 1, comprising selected routinely collected data on emergency treatment and possible subsequent hospital stay from 16 EDs (data source 1: hospital data), merged with the data of these patients from their routine outpatient health care for the period from 2 years before to 1 year after their ED stay (data source 2: outpatient care data) (15, 16).

Scenario 2 utilizes the data for all patients in Germany with statutory health insurance and an outpatient ED visit in 2016 from the collection of nationwide outpatient billing data of the Associations of Statutory Health Insurance Physicians (KV: Kassenärztliche Vereinigungen). Since outpatient treatment of all statutory health insurance companies are invoiced via the KV, including outpatient ED treatment (17), scenario 2 will allow the nation-wide representativeness of scenario 1 to be assessed.

Similarly to scenario 2, as another independent and parallel analysis, scenario 3 will analyze the outpatient and inpatient care before and after an emergency treatment in 2016 using routine data from a system of eleven regional health care funds of AOK (Allgemeine Ortskrankenkassen, statutory health insurance companies), that insure more than 26 million people in Germany (data source 3: AOK routine data).

The present publication focuses on scenario 1.

In order to achieve the goals of INDEED scenario 1, inclusion of all eligible patients is crucial to guarantee representativeness of the study population. Treatment in an ED is generally carried out in an exceptional situation; in some cases patients are not able to give consent due to urgency of treatment and the short treatment period. Therefore, individual declarations of consent are/were not intended in the context of this retrospective secondary use of routine data for INDEED. Instead, data processing had to be based on a statutory norm of consent, requiring complex technical and organizational measures as described below.

### Study Population

EDs were recruited by approaching clinics from an existing ED-network, AKTIN (18), as well as at scientific events of the Emergency Medicine societies in Germany. We intended to recruit hospitals of different sizes throughout Germany. The main requirement for participation was the availability of electronic documentation in the ED in 2016. Data protection regulations in the respective federal states were also taken into consideration and three categories of feasibility were assigned to the federal states of Germany (green, yellow, red). It was examined whether there was a legal norm of permission at the state level to conduct the project without prior informed consent of patients (green−7 times assigned) and which requirements, if any, were associated with this (yellow−3 times). In the absence of such or significant restrictions, the project was assigned to the red category (6 times). Regardless of the categorization, it should be noted that in each case a considerable great effort of argumentation was done to obtain the necessary approvals.

Patients in the participating EDs were included if they had at least one ED visit in 2016 and were insured in one of the existing German statutory health insurance companies, which cover about 87% of the population in Germany (19). We exclude patients with private health insurance as well as cases that are billed via the German Social Accident Insurance. The latter covers accidents at work or recognized occupational diseases. Although they constitute a relevant proportion of cases in German EDs, they are covered by a different regulatory framework. Minimum age has to be 20 years on January 1st 2016, as this corresponds to a minimum age of 18 years at the beginning of our observation period (January 1st, 2014) in the outpatient care data.

**Figure 2:**
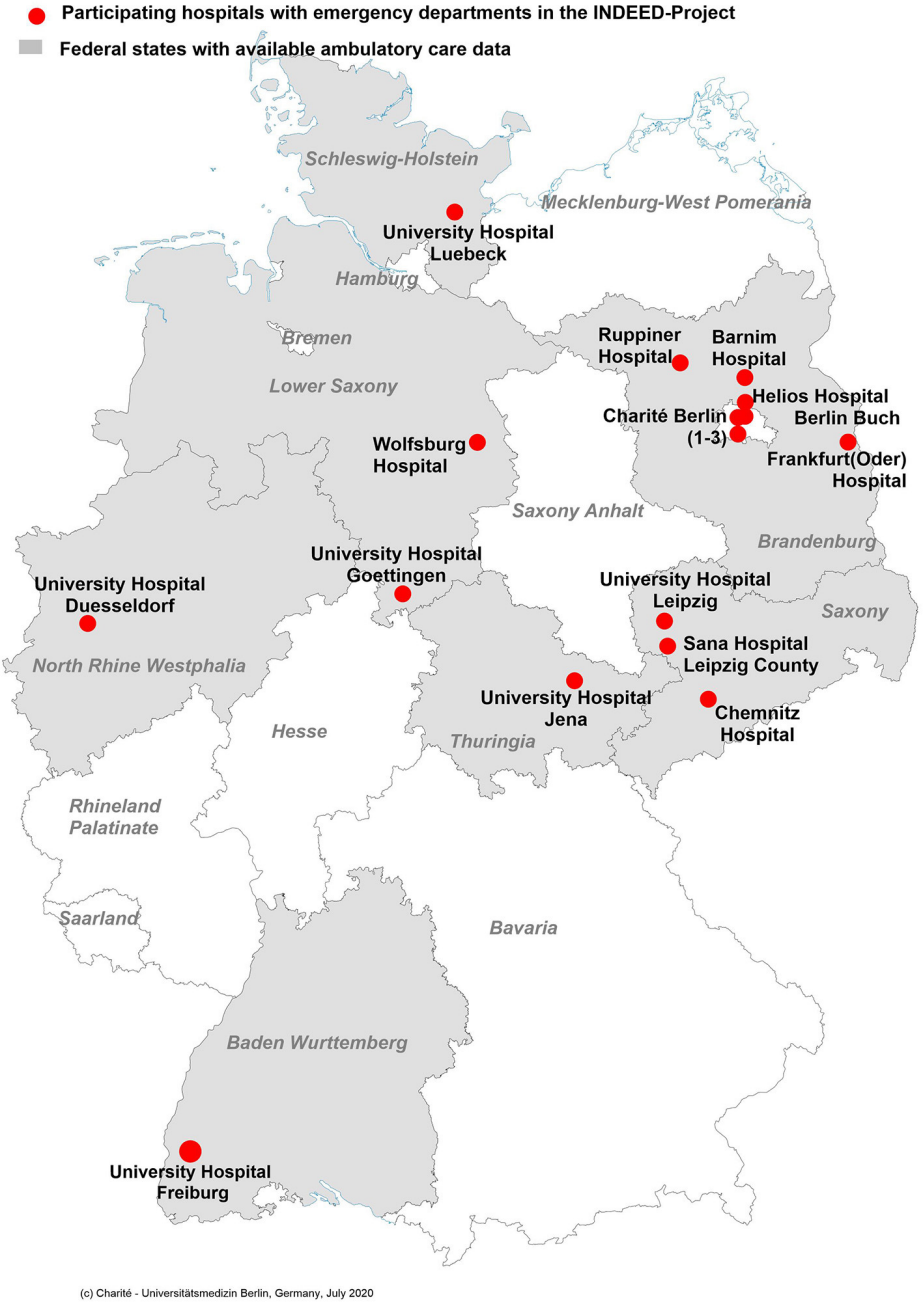
Overview of the 16 participating hospitals with emergency departments (red dots) and the German federal states (in gray) where the regional Associations of Statutory Health Insurance Physicians provided outpatient health care data. “Charité Berlin (1–3)” designated 1 = Campus Virchow Klinikum, 2 = Campus Mitte, 3 = Campus Benjamin Franklin.

The final study population for scenario 1 are ED patients originating from 16 participating EDs in Germany (Figure 2). In addition, basic data of ED and hospital structures are collected according to standards used in previous projects such as the German DGINA (German society for interdisciplinary emergency and acute medicine) network (20), to allow a basic description of the participating EDs and hospitals.

### Data Sources, Data Flow, and Management

In scenario 1, data from two different sources will be merged. Firstly, ED and inpatient data from 2016 will be extracted from 16 hospitals with EDs across Germany. This will include general information about the patient, ED treatment data, vital signs, blood parameters, and data from a subsequent inpatient stay. Secondly, for the time period from 2014 to 2017 outpatient care data will be extracted for all patients who were treated at least once in 2016 in one of the participating EDs. This data will be provided by the Zi after collection from the regional Associations of Statutory Health Insurance Physicians (KV) of the federal states corresponding to the location of the participating EDs. These data sets will include general information about the patient, information on the medical practice and practitioner, diagnoses, performed procedures and their costs, and information about the medication and their costs.

Clinical data usually lack sufficient standardization due to different software systems used for documentation in the EDs, different treatment pathways, different routines and situations, with an additional lack of time for sufficient documentation in emergency situations. The degree of heterogeneity and need for further homogenization and data-coarsening will be determined after data extraction from all hospitals has been completed. Data processing will take place at the INDEED Central Data Management (CDM) office. The list of variables to be extracted from the hospitals and the respective outpatient care data are presented in Supplementary Table 1.

**Figure 3:**
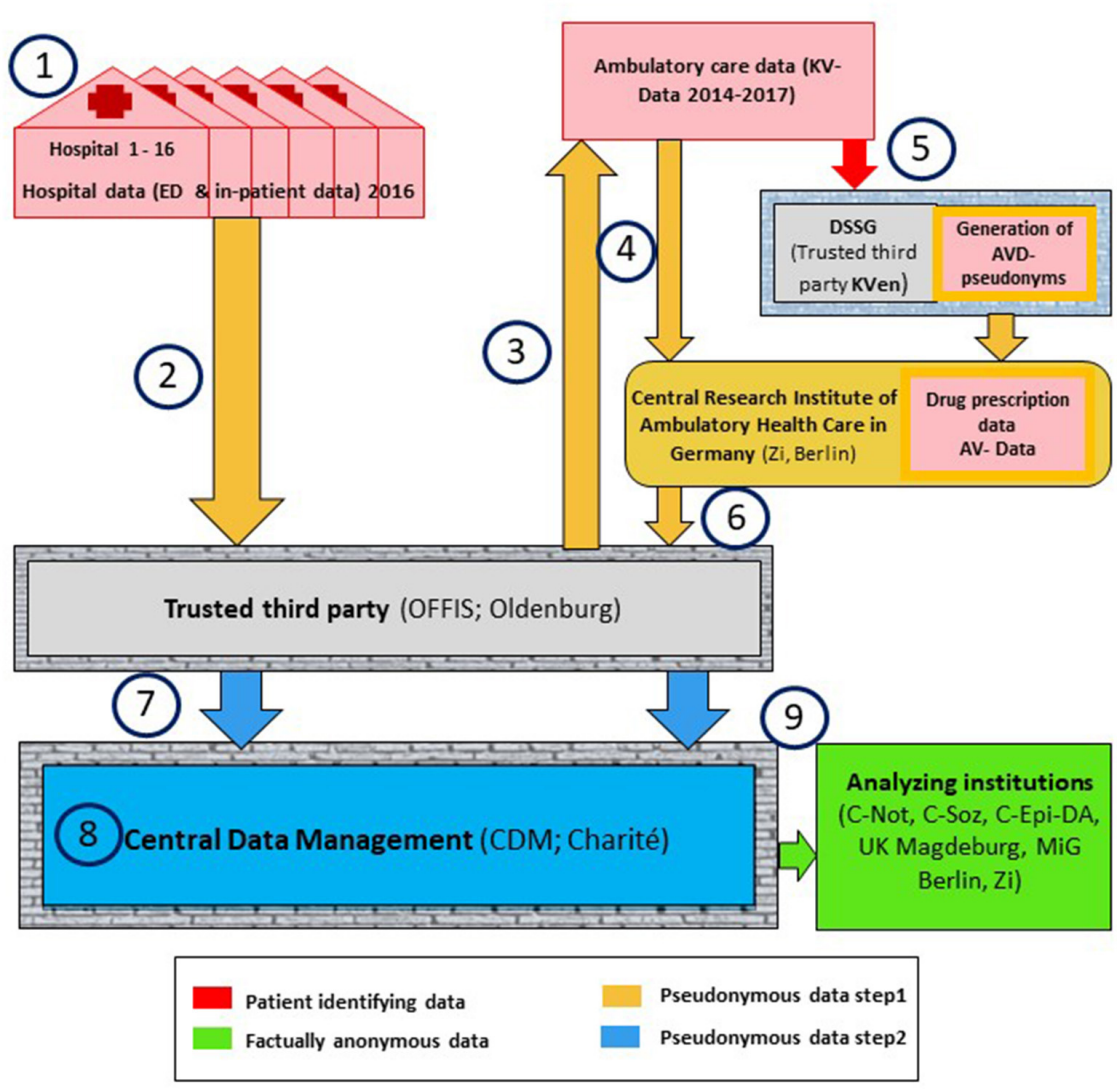
Schematic overview of the data flow in INDEED scenario 1. ED, emergency department; OFFIS, Institute for Information Technology; Oldenburg, trusted third party of the project data; CDM, Central Data Management; KV-data, the outpatient care data originating from the KV; DSSG, Service & Support GmbH (KV data trusted third party); AV-data, medical prescription data; C-Not, Emergency and Acute Medicine (CVK, CCM); Charité, Universitätsmedizin Berlin; C-Soz, Institute of Medical Sociology and Rehabilitation Science; Charité, Universitätsmedizin Berlin; C-Epi-DA, Institute for Social Medicine, Epidemiology and Health Economics; Charité, Universitätsmedizin Berlin, Data analizes; UK Magdeburg, Department of Trauma Surgery; Otto von Guericke University Magdeburg, MiG Berlin, Dept. Health Care Management, Berlin University of Technology; Zi, Central Research Institute of Ambulatory/Outpatient Health Care in Germany.

The data handling procedures from the local hospital queries, the linkage of both data sources (hospital and outpatient care data), up to the final dataset are quite complex: challenges include (i) extracting data from different documentation systems, (ii) cleaning and harmonizing them, and (iii) linking these two data sources (hospital data and outpatient care data) at the patient level, all while conforming to the different and complex data protection rules (Figure 3). All processes regarding data linkage follow and adhere to the “Good Practice Data Linkage” (21).

The data flow (step 1 to 9) is described in more detail in the following section. All data containers are signed and RSA-4096-encrypted (special encryption method named after Rivest, Shamir and Adleman) and all data transfers are handled via SSH/TSL, secured by encryption on transfer level and receiver oriented encryption on file level (22).

#### Extraction and Pseudonymization of Hospital Data

For the linkage of the two data sources (hospital and outpatient care data), an identification number is needed that is unique for each patient and available in both data sources (Figure 3, Step 1). This linking will be based primarily on the electronic health insurance card number (eGK-Nr) and, secondarily, on a combination of surname, first name, and date of birth (Name-DOB; DOB: date of birth) if the eGK-Nr is missing or erroneous. However, in the end it only worked for the first strategy based on the eGK-Nr, due to software issues and lack of time to fix this problem. Of the final 353.926 ED-patients, 290.883 (82.2%) had a valid eGK number, could be identified in the KV data and successfully linked. The eGK-Nr, Name-DOB and the internal hospital case numbers (Case-Nr) for each ED patient visit compose the patients' Identifying Data (IDAT). A computer software, which was specifically developed for the INDEED-project by OFFIS, pseudonymizes these IDAT after final data extraction using a cryptographic hash function. The data set containing the IDAT, the Medical information user Data (MDAT) and an added line identifier (L-ID) is split into separate IDAT and MDAT datasets, with the L-ID in both, and consequently into different encrypted containers. The MDAT include all the information regarding treatment during the ED visit and the possible subsequent inpatient stay. The MDAT can only be decrypted in CDM. Since hospital data will be obtained from different documentation systems within each hospital the above process must be performed on numerous data sets for each hospital. Although these numerous data sets could potentially be merged on-site with the Case-Nr it will be performed in CDM at a later stage, to ensure the highest data quality.

#### Transfer of Hospital Data

The hospital data will be securely transferred and stored on a server at OFFIS (Figure 3, Step 2). A random center-specific number will be assigned to each hospital, and this allocation is only known by OFFIS. This number will allow center-specific adjustments in the statistical analyses. However, the consortium agreement prohibits comparative center-specific analyses.

#### Preparation of Data Linkage at OFFIS: Hospital Data

OFFIS will create a list of INDEED patients based on the pseudonymized eGK-Nr and Name-DOB in the IDAT, additionally using the Case-Nr if inconsistencies occur (Figure 3, Step 3).

#### Selection of Outpatient Care Data and Data Transfer to the Zi

The list of pseudonyms will be imported into the INDEED-software and applied to the outpatient care data from 2014 to 2017 of the KV in the relevant regions (Figure 3, Step 4). Patients who are included in INDEED (i.e., having at least one visit in one of the cooperating EDs in 2016) will be extracted from this data. Then, a separation of the MDAT and pseudonymized IDAT and a subsequent encryption will be applied as per the hospital data.

#### Generation of the Medical Prescription Data Pseudonym at the Trusted Third Party of the KV and Medical Prescription Data Selection at the Zi

For the medical prescription data, the INDEED patients will be pseudonymized in a separate process by the KV trusted third party (Figure 3, Step 5). The Zi who already possesses the medical prescription data and the corresponding pseudonyms created by the trusted third party will then extract the INDEED patient's medical prescription data and merge it with the KV data.

#### Data Transfer of KV and Medical Prescription Data to OFFIS

In the next step, the Zi transfers KV and medical prescription data (from steps 4 and 5) of the INDEED-patients to OFFIS (Figure 3, Step 6). The IDAT can only be decrypted by OFFIS, the MDAT can only be decrypted in CDM.

#### Data Linkage (OFFIS) and Data Transfer to Central Data Management

The patient pseudonyms within the IDAT (eGK-Nr and Name-DOB) for both the KV and medical prescription data as well as the hospital data are then replaced with a new single pseudonym at OFFIS, to produce the INDEED patient number (I-PNr) (Figure 3, Step 7). Together with the MDAT, these IDAT are then transferred to CDM.

#### Data Processing (Data Management, Cleaning, Harmonization, Plausibility, Etc.)

Each variable for each hospital will be cleaned (e.g., irrelevant text removed, data formats standardized) and homogenized (e.g., standardized units of measurement and missing value symbols) (Figure 3, Step 8). Variables with varying response categories between hospitals will be harmonized using standardized categories determined by experienced clinicians and methodologists of the consortium, who will also determine plausibility rules (e.g., min/max cut-offs, logical data values) and the rules for implausible values (including exclusion).

#### Database Structure and Data Provision to the Analyzing Institutions

Due to its structure and size, data will be stored in a relational database and CDM will provide partitions of the processed data to the analyzing partner institutions within the consortium upon request (Figure 3, Step 9). In accordance with the consortium's agreement, the partner institution will initiate this process by informing the management board (MB) about the proposed research question(s) to be analyzed and the respective data needed. The MB will decide if the research question is appropriate under the general INDEED scopes, taking other partner's research areas into account. Afterwards, the analyzing partner officially requests the data from the MB's Data-Use-And-Access-Committee (DUAC). The DUAC checks whether the requested variables are suitable for answering the research questions. Upon approval, the DUAC will inform CDM, who will then prepare the relevant data set. Before release of the data, CDM will generate new random ID-numbers for each specific analysis, replacing the identifying variables originating from the IDAT (e.g., I-PNr, Case-Nr) to ensure that the partners will be provided only with factually anonymized data. In addition, data-coarsening may take place before data release for variables that may potentially be used to identify an individual person (e.g., by categorizing variables into broader classes, or by eliminating or categorizing extreme values).

### Sample Size Estimation and Statistical Analyses

We assumed that 15 to 20 EDs across Germany will result in a sample of EDs yielding different hospital structures and locations. We were assuming that these EDs have an average of about 34,000 cases per year. This assumption is based on a general estimate of the average number of cases in German EDs (3, 23, 24), and yields an expected total number of 510,000 to 680,000 cases. However, since about 15% of patients have multiple emergency contacts per year (based on ED data from Charité in Berlin), the estimated total number of patients is around 442,000 to 589,000. Applying the study's inclusion criteria (e.g., adult patients with statutory health insurance) will limit the number of eligible patients to about 86.5% of the total population in Germany in 2016 (24). Thus, we expect a total of approximately 400,000 ED patients to be available for our data analysis.

With this sample size, precise estimates of prevalences are possible even within small subgroups: for example, in any given subgroup of 1% of the total number of patients (i.e., approximately 4,000 patients) a prevalence of 50% could be estimated with a precision of ±1.6% (half width of a two-sided exact 95% confidence interval). In this example, a prevalence of 50% was chosen, as this proportion shows the greatest variation. A prevalence higher or lower than 50%, or subgroups larger than 1%, would lead to narrower confidence intervals, i.e., more accurate estimates.

### Data Protection and Ethical Aspects

Linking health care data on an individual level requires a high effort of data protection and data security as well as compliance with ethical standards. For the project the ethical principles of the Helsinki Declaration will be taken into account (25, 26) and the legally compliant handling of medical treatment data and personal data will be ensured.

The study was approved by the ethics committee of the Charité–Universitätsmedizin Berlin (application: EA4/086/17). The data protection concept applies to all linked datasets and considers the corresponding flow, time and scope of data linkage and data analyses, was approved by the TMF Working Group of Data Protection on the 14th February 2018 as well as by the institutional data protection officer at Charité–Universitätsmedizin Berlin.

The legal context of collecting routine treatment data for each hospital depends upon whether it is a public or private entity (27). In addition, both federal and state data regulations have to be considered. The legal framework in Germany for social data has been set out in the Social Code Books, especially SGB X, V und I. The social data of the KV are especially protected (§ 35 SGB I) and data transfer must be approved according to the legal regulations of paragraphs §§67a ff. SGB X. The basis for transferring social data for the present research project is paragraph § 75, Section 1, number 1, SGB X (research projects on the basis of social data) and requires approval by the responsible regulatory authority (VfD_INDEED_17_003844; NCT03224078).

The INDEED project received all necessary approvals from the responsible regulatory authorities, before the respective hospital data extraction was started. The last approval was granted by June 2019. For data protection, this included 16 approvals from the data protection officers of the participating hospitals, two approvals from the data protection authorities of the federal states as well as eight approvals from the responsible regulatory authorities for social data.

## Expected Results and Status Quo

The central research question is to identify and characterize patients with an adequate, inadequate, or avoidable ED utilization. Further research questions include: Which outpatient care did patients receive before and after their stay in an ED and what are influencing factors of ED utilization? An additional focus will be put on the analysis of vulnerable subgroups (e.g., multimorbid patients, elderly patients). The results have the potential to contribute to the development of health policy innovations and interventions for need-based, purposeful, and economic adjustment of care processes and structures. Identified patient clusters shall be used to adjust health care across sectoral boarders.

The methodological aim of INDEED is to create an infrastructure that allows the linkage and use of routinely collected ED and hospital data in combination with routine outpatient health care data. This includes the development and implementation of a sustainable/generic data protection concept, the standardization of emergency care data across varying hospital information systems and the identification of routinely collected key characteristics of the outpatient sector. A main challenge will be the linkage of individual patient data from different care providers based on pseudonyms. Compared to other studies on EDs, we are able to include patients across outpatient and inpatient care from different German statutory health insurance funds. Previous studies focused either on analyzing data from a single disease (28) or investigated German wide emergency data separately for the inpatient and outpatient sector (4).

All 16 EDs have completed the data extraction process by September 2019 and their data has been transferred in double pseudonymized form to the CDM. The data included general information about the patient, ED treatment data and data of the following in-patient stay. The availability of the data, its characteristics and its quality was heterogeneous and varied between EDs. A high level of data processing is required, which is currently at an advanced stage. The last data set from the KV was transferred to CDM at the beginning of November 2020. The linking of this ED-data with the outpatient care data is work in progress and a merged data set for analysis is expected by the end of 2020.

International studies have shown that the linkage of existing routine data enables cross-sectoral and interdisciplinary health services research and can thus form the basis for interventions in health care (29, 30). In Germany, research usually focuses on the analysis of health care situations within individual sectors or on patients with specific diagnoses. Hence, the results are limited in their informative value for emergencies in which the focus is not on diagnosis but on symptoms (8). The linkage of ED data and routine outpatient health care data at the individual level has so far not been conducted in Germany.

However, routine data has been linked both nationally and internationally for the analysis of cross-sectoral care in settings differing from our project, helping to provide a less distorted view of the reality of medical care provision. A few cross-sectional and longitudinal analyses have been conducted, but not related with emergency care treatment (31, 32). Such analyses counterbalance some of the disadvantages of surveys, such as the recruitment of hard-to-reach patient groups, selection, and recall bias.

The hospitals participating in INDEED are institutions with long established structures and the responsible staff have previously been active in emergency medicine research. Some hospitals interested in INDEED were not able to participate due to limited resources or personnel, insufficient IT support capacity, lack of electronic documentation or access to it, or a change of hospital contact person. An especially challenging obstacle was the specific responsibility of each federal state in Germany regarding data protection regulation. For example, the Bavarian state hospital law does not allow non-anonymized data to leave the hospital. Since the aim of the INDEED project is to link hospital data with outpatient health care data, we were not able to include any interested hospitals from Bavaria.

Although we included 16 hospitals from across Germany, we did not randomly choose them and do not consider this selection as being representative for Germany. Our findings can therefore only be interpreted in a local context, i.e., the catchment area of the hospital. However, since we will include data from various types and a considerable number of university and non-university hospitals in different federal states, we expect to obtain a good picture of the cross-sectoral pathways of ED patients in the German health care system.

A further limitation of INDEED is the linkage of hospital data with outpatient treatment data held by the regional Associations of Statutory Health Insurance Physicians, since there are also privately billed health services that do not appear in these data. Furthermore, treatments reimbursed by the German Social Accident Insurance, i.e., in connection with an occupational accident, are not included either. Future research projects should also address these subgroups.

One strength of INDEED are the three scenarios using different large data evaluation approaches. The representativeness of the included patients and the results from the INDEED scenario 1 cohort will be checked by comparing them with specific characteristics of the other large cohorts from scenarios 2 and 3, whose data originates from the Associations of Statutory Health Insurance Physicians and the AOK.

The extraction of pseudonymous data without consent from the hospitals requires a very comprehensive data protection concept. This was prepared under the leadership and with the specific technological and methodological expertise of the TMF as a consortium partner. The concept was thoroughly checked and discussed by the multidisciplinary working group of the TMF for data protection and subsequently approved by this group. It was then made available to all the local data protection authorities of the hospitals. In Thuringia and Brandenburg it was necessary to obtain additional and explicit approval from the federal state data protection authorities, a time-consuming process, which had not been expected upon application and after the first experiences with other federal states. The data protection concept for the outpatient treatment data from the Associations of Statutory Health Insurance Physicians was designed by the consortium partner Zi, the central research institute for this specific outpatient health care data. They transferred the application and the concept to the respective eight regional Associations of Statutory Health Insurance Physicians, which had to obtain the relevant approvals from the federal state authorities as well. This resulted in further long and bureaucratic processes lasting between several months to over a year.

## Data Availability Statement

Due to the very high sensitivity of the data in the project, it is not possible to make them available to the public. In the central data management of the project the data are already pseudonymised twice. They are made available exclusively to the analyzing partners of the consortium in anonymised form and only with variables that are matched to the research question.

## Ethics Statement

The studies involving human participants were reviewed and approved by Ethikkommission der Charité-Universitätsmedizin Berlin/Germany. Written informed consent for participation was not required for this study in accordance with the national legislation and the institutional requirements.

## Author Contributions

CL was involved in creation of a new software used for the project. All authors made substantial contributions to the conception of the project and have drafted the publication.

## Conflict of Interest

The authors declare that the research was conducted in the absence of any commercial or financial relationships that could be construed as a potential conflict of interest.

## Abbreviations

AV-data: medical prescription data
CDM: Central Data Management
Case-Nr: the internal hospital Case Numbers for each ED patient visit
DSSG: Service & Support GmbH (KV data trusted third party)
eGK-Nr′: electronic health insurance card number
ED: emergency department
IDAT: person Identifying Data
I-PNr: INDEED patient number (created in second stage pseudonymization, i.e., pseudonymization of the pseudonymized eGK-Nr and Name-DOB)
INDEED: Utilization and cross-sectoral patterns of care for patients admitted to emergency departments in Germany
KV: Association(s) of Statutory Health Insurance Physicians (responsible for regions that correspond mostly to the federal states in Germany)
KV-data: the outpatient care data originating from the KV
L-ID: line identifier used to link the IDAT and MDAT in CDM
MDAT: Medical information user Data
MiG: Department of Health Care Management of the Berlin University of Technology
Name-DOB: a combination of family name, first name and date of birth
OFFIS: Institute for Information Technology, Oldenburg–trusted third party of the project data
TMF: Technology, Methods, and Infrastructure for Networked Medical Research
WIdO: AOK Research Institute, Federal Association of AOK, Berlin, Germany
UKMD: Department of Trauma Surgery, Otto von Guericke University Magdeburg, Magdeburg, Germany
Zi: Central Research Institute of Ambulatory/Outpatient Health Care in Germany, Berlin, Germany.
