# Supplementary material for: INDEED–Utilization and Cross-Sectoral Patterns of Care for Patients Admitted to Emergency Departments in Germany: Rationale and Study Design
Source: Front Public Health. 2021 Apr 16;9:616857. doi: 10.3389/fpubh.2021.616857 (PMC8085405; doi:10.3389/fpubh.2021.616857)
Supplement: Supplementary file 1 [file Table_1.DOCX]

Supplementary Material

# Supplementary Table 1

Table S1: variables collected for INDEED project scenario 1/data source 1.

| **1a) Data from the hospital** (ED and subsequent inpatient stay) - General information |
| --- |
| eGK number = electronic health insurance card number *(used only for creation of pseudonym to enable data linkage)* |
| Family name *(used only for creation of pseudonym to enable data linkage)* |
| First name *(used only for creation of pseudonym to enable data linkage)* |
| Date of birth *(used only for creation of pseudonym to enable data linkage)* |
| Internal hospital case number (will be pseudonymized) |
| Age |
| Sex |
| Nationality |
| Statutory health insurance company |
| Institutional number of the health insurance company |
| Postal code (first two numbers) of residence |
| **1b) Data from the hospital** (ED and subsequent inpatient stay) - Treatment in the emergency department (ED) |
| Transport method to the hospital |
| Time of arrival |
| Triage category / acuity level |
| Time of triage |
| Main symptom/sign of complaint |
| Time of first contact with physician |
| Time when treatment was ended |
| Time of leaving the ED |
| Case type: out-patient / in-patient / other |
| Medical imaging performed (X-ray/ magnetic resonance imaging/ computer tomography/ sonography) |
| Urine examination performed |
| Electrocardiogram performed |
| Treating medical specialty in the ED |
| Number of different hospital departments/ wards visited |
| All documented diagnoses in the ED (ICD Codes) |
| Level of confidence in the ED diagnoses (suspicion/ excluded/ confirmed/ status post event) |
| Transfer: internal/ external/ discharged/ deceased |
| **1c) Data from the hospital** (ED and subsequent inpatient stay) - Vital signs, scores und blood parameters in the ED |
| Breathing frequency, respiratory rate |
| Heart rate |
| Blood pressure (systolic, diastolic) |
| Oxygen saturation |
| Body temperature |
| Pain level (numerical rating scale, 0 (none) - 10 (worst pain) |
| Glasgow Coma Scale |
| Time when blood was sample drawn |
| Blood test results (C-reactive protein, red blood cell distribution width, creatinine, lactate, troponin, thyrotropin) |
| **1c) Data from the hospital** (ED and subsequent inpatient stay) - Subsequent inpatient stay |
| Reasons for admission |
| Ward department (admission ward/ discharge ward) |
| Time of hospital admission |
| Time of hospital discharge |
| Admission diagnosis |
| Main diagnosis of hospital stay (ICD Code) |
| Secondary diagnoses (ICD Code) (1-x) |
| Procedures (OPS Code, Operation and Procedure Classification System) (1-x) |
| Time of procedures (1-x) |
| Diagnosis Related Groups (DRG) |
| Number of stays in Intensive Care Unit (ICU) |
| Number of hours of artificial respirator |
| Reason for discharge |
| **2a) Ambulatory health care data** - General information |
| Region of the Associations of the statutory health insurance physicians in Germany |
| Quarter of the ambulatory treatment |
| Patient’s year of birth |
| Sex of patient |
| Pseudonym of the medical doctor |
| German statutory health insurance fund number (denotes the health insurance company of the patient) |
| Insurance status (member, family co-insured, retired) |
| Disease management program for: diabetes type 1/2, breast cancer, coronary heart disease, asthma, or chronic obstructive pulmonary disease (COPD) |
| Postal code (in Germany) |
| County/district |
| Federal state of residence (in Germany) |
| **2b) Ambulatory health care data** - Medical practice and practitioner |
| Medical expertise / specialization |
| Type of medical practice according to doctors register |
| Professional classification (General practitioner, medical specialist, ambulatory health care center) |
| **2c) Ambulatory health care data** - Diagnosis |
| Type of treatment case (initial, referral, emergency) |
| Ambulatory diagnosis (ICD Code) |
| Level of confidence in the ambulatory diagnosis |
| Part/area of the body |
| Diagnosis type acute / chronic |
| Level of confidence of the permanent ambulatory diagnosis |
| **2d) Ambulatory health care data** - Performed procedures and their costs |
| Fee schedule item [in German: Gebührenordnungsposition, GOP] |
| Number of fee schedule item |
| Date of fee schedule item |
| Type of treatment case (f.e. treatment as an emergency, in-patient or outpatient treatment, treatment as holiday replacement…) |
| Operation and Procedure Classification System (OPS) code (the German modification of the International Classification of Procedures in Medicine, ICPM) |
| OPS date |
| **2e) Ambulatory health care data** - Medication and their costs |
| Date of prescription |
| Date when medication acquired |
| ATC* classification |
| PZN classification [in German: Pharmazentralnummer] used for medication, assistive technology and other pharmacy products |
| Amount of prescribed drug (according to prescription) |
| Defined Daily Doses (DDD) |
| Provision of medication in emergency service |
| Price in € (Gross) |
